# The role of GH/IGF-1 axis dysfunction and inflammatory cytokines in pediatric short stature

**DOI:** 10.5937/jomb0-57933

**Published:** 2025-11-05

**Authors:** Qingfu Yang, Shanshan Chen, Tieniu Wu, Jingxin Peng, Lidan Mao

**Affiliations:** 1 Ganzhou Maternal and Child Health Hospital, Department of Paediatrics, Ganzhou City, Jiangxi Province, China; 2 The Affiliated Yangming Hospital of Ningbo University (YuYao People's Hospital), Department of Paediatrics, Yuyao City, Zhejiang Province, China

**Keywords:** pediatric short stature, GH/IGF-1 axis, inflammatory cytokines, interleukin-6, interleukin-8, genetic mutations, growth hormone receptor, pediijatrijski niski rast, GH/IGF-1, inflamatorni citokini, interleukin-6, interleukin-8, genetske mutacije, receptor za hormon rasta

## Abstract

**Background:**

Short stature in children is a common clinical condition frequently associated with abnormalities in the GH/IGF-1 axis. Emerging evidence points to the involvement of inflammatory cytokines and serum markers in modulating this dysfunction. This study aims to investigate the molecular pathways underlying GH/IGF-1 axis impairment and assess the levels of inflammatory cytokines and other related biomarkers in children with short stature.

**Methods:**

A total of 150 children diagnosed with short stature were recruited from the endocrinology department of a tertiary care hospital. An ageand sex-matched group of 150 healthy children served as controls for comparison. Serum concentrations of growth hormone (GH), insulin-like growth factor-1 (IGF-1), interleukin-6 (IL-6), interleukin-8 (IL-8), and other relevant biomarkers were measured using enzyme-linked immunosorbent assay (ELISA). Genetic testing was performed to detect mutations in genes involved in the GH/IGF-1 signalling pathway. Data analysis was conducted using SPSS software, with statistical significance set at p&lt; 0.05.

**Results:**

Children with short stature showed significantly reduced GH and IGF-1 levels (p&lt; 0.001) and elevated IL-6 and IL-8 levels (p&lt; 0.01). A moderate negative correlation was found between IL-6 and IGF-1 levels (r=-0.45, p&lt; 0.001), suggesting that inflammation may impair growth signalling. GHR gene mutations were significantly more common in the short stature group (14.7% vs. 2.7%, p&lt; 0.001) and were associated with lower IGF-1 levels.

**Conclusions:**

This findings of the study suggest that impaired GH/IGF-1 signalling, increased inflammatory cytokines, and a higher prevalence of GHR gene mutations collectively contribute to the pathophysiology of pediatric short stature. These results highlight the need for integrative diagnostic approaches and future therapeutic strategies that target both endocrine and inflammatory pathways.

## Abstract (sr)

**Uvod:**

Nizak rast kod dece je često kliničko stanje koje je često povezano sa poremećajima u GH/IGF-1 signalizaciji. Novi dokazi ukazuju na ulogu inflamatornih citokina i serumskih markera u modulaciji ove disfunkcije. Cilj ove studije je da istraži molekularne mehanizme koji stoje iza oštećenja GH/IGF-1 signalizacije i da proceni nivoe inflamatornih citokina i drugih povezanih biomarkera kod dece sa niskim rastom.

**Metode:**

Regrutovano je ukupno 150 dece sa dijagnozom niskog rasta sa odeljenja za endokrinologiju tercijarne bolnice. Grupa od 150 zdrave dece, usklađena po starosti i polu, je korišćena kao kontrolna grupa za upoređivanje. Serumske koncentracije hormona rasta (GH), insulin sličnog faktora rasta-1 (IGF-1), interleukina-6 (IL-6), interleukina-8 (IL-8) i drugih relevantnih biomarkera merene su korišćenjem enzimske imunoanalize (ELISA). Genetska ispitivanja su sprovedena kako bi se detektovale mutacije u genima koji su uključeni u GH/IGF-1 signalizaciju. Analiza podataka je sprovedena korišćenjem SPSS softverskog paketa, a statistička značajnost postavljena je na p&lt;0,05.

**Rezultati:**

Deca sa niskim rastom su pokazala značajno smanjene nivoe GH i IGF-1 (p&lt; 0,001) i povišene nivoe IL-6 i IL-8 (p&lt; 0,01). Pronađena je umerena negativna korelacija između nivoa IL-6 i IGF-1 (r=-0,45, p&lt; 0,001), što sugeriše da upala može da ošteti signalizaciju rasta. Mutacije u GHR genu su bile značajno češće u grupi sa niskim rastom (14,7% naspram 2,7%, p&lt; 0,001) i bile su povezane sa nižim nivoima IGF-1.

**Zaključak:**

Rezultati ove studije sugerišu da oštećena GH/IGF-1 signalizacija, povećani inflamatorni citokini i veća učestalost mutacija u GHR genu zajedno doprinose patofiziologiji pediijatrijskog niskog rasta. Ovi rezultati ističu potrebu za integrativnim dijagnostičkim pristupima i budućim terapijskim strategijama koje ciljaju i endokrine i inflamatorne putanje.

## Introduction

Short stature in children, clinically defined as height below the 3rd percentile for age and sex, is a frequent concern in pediatric practice [1] [2] [3]. While it may occur as an isolated finding, it often reflects broader pathological processes, including genetic disorders, endocrine dysfunction, chronic systemic diseases, or nutritional deficits [4]. Among these, impaired signalling through the growth hormone (GH) and insulin-like growth factor-1 (IGF-1) axis represents one of the most well-characterised mechanisms underlying growth failure [5]. This endocrine pathway regulates somatic growth through a cascade in which pituitary-derived GH stimulates hepatic IGF-1 production, which in turn promotes cellular proliferation in bone and muscle tissues [6]. Disruptions at any point in this axis can lead to clinically significant growth retardation.

Growth hormone deficiency (GHD) exemplifies the classic disorder of this system, marked by insufficient GH secretion or action and consequent reductions in circulating IGF-1 [7] [8]. However, recent research highlights that growth impairment can also arise from other molecular defects, including abnormalities in GH receptor function, post-receptor signalling pathways, or IGF-1 biosynthesis [9]. This complexity underscores the need for a nuanced diagnostic approach, as traditional reliance on serum GH and IGF-1 measurements alone may fail to identify atypical presentations or multifactorial etiologies involving concurrent genetic, metabolic, or inflammatory contributors.

Emerging evidence implicates chronic inflammation as a potential modulator of growth plate dysfunction, with particular interest in the roles of interleukin-6 (IL-6) and interleukin-8 (IL-8) [10]. These cytokines, elevated in various inflammatory states, may exert inhibitory effects on the GH/IGF-1 axis through multiple mechanisms. IL-6 has been shown to suppress pulsatile GH secretion at the hypothalamic-pituitary level [11], while IL-8, primarily recognised for its chemotactic functions, may indirectly impair growth through sustained inflammatory microenvironments [12]. Such findings are clinically relevant, as children with chronic inflammatory conditions (e.g., juvenile idiopathic arthritis, inflammatory bowel disease, thyroid disorders) frequently exhibit growth retardation disproportionate to their primary disease severity [13] [14] [15] [16].

The correlation between inflammation and endocrine dysfunction presents a compelling area for investigation, particularly given observations that elevated inflammatory markers correlate with blunted GH responses in short-stature populations [17] [18]. While the suppressive effects of cytokines on GH secretion are well-documented in vitro, the in vivo pathways linking systemic inflammation to growth plate biology remain incompletely characterised. This knowledge gap is particularly salient in idiopathic short stature, where subclinical inflammation may contribute to unexplained growth failure.

Parallel to these findings, advances in genetic diagnostics have identified numerous monogenic causes of growth impairment. Pathogenic variants in the GH receptor (GHR) gene, for instance, can cause GH insensitivity syndromes through impaired receptor dimerisation or downstream signalling. Similarly, mutations affecting IGF-1 synthesis or bioavailability demonstrate the critical role of this pathway in postnatal growth. These discoveries have refined the classification of growth disorders while revealing substantial phenotypic variability even among individuals with identical genetic defects - a phenomenon potentially explained by modifier genes or environmental interactions [19].

Despite these advances, critical questions persist regarding the interplay between inflammatory mediators, endocrine pathways, and genetic susceptibility in childhood growth failure. Current research often examines these factors in isolation, neglecting potential synergies that could explain clinical heterogeneity. Moreover, few studies have systematically evaluated whether cytokine profiles or genetic polymorphisms predict therapeutic responses to GH treatment - a clinically significant omission given the variable efficacy of such interventions.

This study aims to address these gaps through a comprehensive analysis of GH/IGF-1 axis dysfunction, inflammatory biomarkers (with emphasis on IL-6 and IL-8), and GHR mutation status in a pediatric cohort with short stature. By employing comparative analyses with healthy controls, we seek to (1) characterise distinct molecular signatures associated with growth impairment, (2) explore interactions between inflammatory and endocrine pathways, and (3) assess the predictive value of genetic testing in therapeutic decision-making. These insights may inform more personalised management strategies while identifying novel targets for pharmacological intervention in treatment-resistant cases.

## Materials and methods

### Study design and participants

This investigation employed a cross-sectional design and was carried out at a pediatric endocrinology clinic. Its primary objective was to examine the molecular mechanisms behind short stature in children, with an emphasis on the disruption of the growth hormone (GH)/insulin-like growth factor-1 (IGF-1) pathway, inflammatory markers, and genetic mutations. The study included 150 children diagnosed with short stature, compared against a control group of 150 healthy children matched for age and sex.

Children diagnosed with short stature were recruited from the clinic. They were identified based on clinical criteria, specifically a height measurement falling below the 3rd percentile for their age and sex, as indicated by growth charts. Eligibility for the study required participants to be between the ages of 2 and 18, meeting the short stature criteria without a history of chronic conditions such as asthma, diabetes, or genetic diseases. Furthermore, these children could not have undergone any growth hormone treatments or be on medications that could affect growth. Exclusion criteria encompassed secondary causes of short stature, including malnutrition and other endocrine-related disorders.

The control group consisted of 150 healthy children who were also matched for age, sex, and socioeconomic status. These children, assessed during routine health check-ups, exhibited normal growth patterns, with height and weight within the 25th to 75th percentiles, and had no known medical or endocrine disorders.

### Ethical considerations

The study received approval from the hospital's institutional review board (IRB), and all procedures followed the ethical guidelines outlined in the Declaration of Helsinki. Informed consent was obtained from the parents or legal guardians of all participants, and children aged 7 years and older also gave their assent. Before enrollment, all participants were thoroughly informed about the study's purpose, potential risks, and benefits.

### Sample collection and laboratory procedures

Blood samples were collected in the early morning after an overnight fast to reduce the influence of recent food intake on biomarker levels. Venous blood was drawn into serum separator tubes (SST), left to clot at room temperature for 30 minutes, and then centrifuged at 3,000 rpm for 10 minutes to isolate the serum. The serum samples were promptly stored at -80°C until further analysis.

Serum concentrations of growth hormone (GH) and insulin-like growth factor-1 (IGF-1) were measured using enzyme-linked immunosorbent assay (ELISA) kits. The GH assay had a detection sensitivity of 0.1 ng/mL, while the IGF-1 assay had a sensitivity of 10 ng/mL. Both assays demonstrated intra- and inter-assay coefficients of variation below 10%, ensuring reliable results.

Inflammatory cytokines were also assessed using ELISA:

- IL-6 levels were measured with a sensitivity of 3 pg/mL.
- IL-8 levels were measured with a sensitivity of 5 pg/mL.

All measurements were performed in duplicate to enhance accuracy. In addition, C-reactive protein (CRP) levels were evaluated using an immunoturbidi-metric assay, and serum albumin concentrations were determined via the bromocresol green dye-binding method to assess systemic inflammation further.

### Genetic analysis

For the genetic analysis, DNA was isolated from peripheral blood leukocytes using a standard extraction kit. The analysis focused on detecting mutations in the growth hormone receptor (GHR) gene. Specific exons of the GHR gene were amplified using polymerase chain reaction (PCR), and the amplified products were subsequently subjected to Sanger sequencing. The resulting sequences were analysed to identify any mutations or polymorphisms. The frequency of these genetic variations was then compared between the short-stature group and the control group.

### Data quality control

All laboratory analyses were performed in duplicate, and the mean value was used to minimise errors in measurement. Laboratory personnel were blinded to the group assignments to ensure objectivity. To ensure consistency, a subset of serum samples was re-analysed by a second technician, and any discrepancies were addressed through a review of raw data and re-testing when necessary.

### Statistical analysis

Data were analysed using SPSS (version 26). Continuous variables were reported as means±standard deviations and categorical variables as frequencies and percentages. Group comparisons were made using independent t-tests or Mann-Whitney U tests for continuous data and chi-square or Fisher's exact tests for categorical data. Correlations between IL-6, IGF-1, and other markers were assessed using Pearson's or Spearman's correlation based on data distribution. A p-value<0.05 was considered statistically significant. GHR gene mutation frequencies were compared between groups using Fisher's exact test.

## Results

### Demographic and baseline characteristics

The study included 150 children with short stature and 150 healthy controls, matched by age and sex. A summary of the demographic characteristics of the participants is presented in Table 1. The average age of children in the short-stature group was 8.5±3.4 years, while the control group had an average age of 8.7±3.3 years, with no statistically significant difference between the two groups (p=0.63). The short-stature group consisted of 80 boys (53.3%) and 70 girls (46.7%), while the control group included 78 boys (52%) and 72 girls (48%), with no significant difference in gender distribution between the groups (p = 0.79).

**Table 1:** Demographic and baseline characteristics.

Characteristic	Short Stature	Control Group	p-value
Age (years)	8.5±3.4	8.7±3.3	0.63
Sex (Male, n%)	80 (53.3%)	78 (52%)	0.79
Sex (Female, n%)	70 (46.7%)	72 (48%)	0.79

### Serum marker levels

Significant differences were observed in the serum levels of growth hormone (GH), insulin-like growth factor-1 (IGF-1), inflammatory cytokines (IL-6 and IL-8), and other markers such as C-reactive protein (CRP) and serum albumin between the short-stature and control groups. The short stature group had a mean GH level of 0.72±0.43 ng/mL, which was lower than the control group's mean GH level of 2.88±1.12 ng/mL (p<0.01). Similarly, the short stature group had a mean IGF-1 level of 113.6±32.5 ng/mL, which was significantly lower than the control group's mean of 224.5±56.3 ng/mL (p<0.001).

Inflammatory cytokine levels were also notably higher in the short-stature group. The mean IL-6 level in the short stature group was 18.2±7.3 pg/mL, significantly elevated compared to the control group's mean of 7.4±3.1 pg/mL (p<0.01). Similarly, iL-8 levels in the short-stature group were significantly higher, with a mean of 134.5±44.6 pg/mL, compared to 68.9±27.3 pg/mL in the control group (p<0.01). Table 2

**Table 2:** Serum marker levels.

Parameter	Short Stature<br>Group (n = 150)	Control<br>Group<br>(n = 150)	p-value
Growth<br>Hormone (GH)<br>(ng/mL)	0.72±0.43	2.88±1.12	<0.001
Insulin-Like<br>Growth Factor 1<br>(IGF-1) (ng/mL)	113.6±32.5	224.5±56.3	<0.001
Interleukin-6 (IL-6)<br>(pg/mL)	18.2±7.3	7.4±3.1	<0.01
Interleukin-8 (IL-8)<br>(pg/mL)	134.5±44.6	68.9±27.3	<0.01
C-Reactive<br>Protein (CRP)<br>(mg/L)	5.1±2.2	1.3±0.8	<0.001
Serum<br>Albumin<br>(g/dL)	3.6±0.5	4.2±0.4	<0.001

Additionally, CRP levels were significantly elevated in the short stature group (5.1±2.2 mg/L) compared to the control group (1.3±0.8 mg/L, p<0.001), while serum albumin levels were significantly lower in the short stature group (3.6±0.5 g/dL) compared to the control group (4.2±0.4 g/dL, p<0.001).

### Genetic analysis

Genetic analysis of the growth hormone receptor (GHR) gene showed higher prevalence of mutations in the short stature group compared to the control group. Of the 150 children with short stature, 22 (14.7%) exhibited mutations in the GHR gene, whereas only 4 (2.7%) children in the control group had similar mutations (p<0.001). These mutations were predominantly located in exons 6 and 7 of the GHR gene, which are essential for GH binding and receptor functionality. The presence of these mutations was linked to significantly lower serum IGF-1 levels in the affected children. In children with GHR mutations, the mean IGF-1 level was 97.3±29.5 ng/mL, significantly lower than the mean IGF-1 level of 145.2±41.4 ng/mL in children without GHR mutations (p<0.01). This finding suggests that GHR gene mutations may play a role in growth impairment by decreasing IGF-1 production. Table 3

**Table 3:** Comparison of GHR gene mutations and IGF-1 levels between groups.

Group	GHR Mutation<br>Present<br>(n, %)	GHR Mutation<br>Absent<br>(n, %)	p-value<br>(Mutation<br>Frequency)	Mean IGF-1<br>(ng/mL) GHR<br>Mutation Present	Mean IGF-1<br>(ng/mL) GHR<br>Mutation Absent	p-value<br>(IGF-1 Levels)
Short Stature<br>(n = 150)	22 (14.7%)	128 (85.3%)	<0.001	97.3±29.5	145.2±41.4	<0.01
Control<br>(n = 150)	4 (2.7%)	146 (97.3%)		—	—	—

### Comparison of growth hormone treatment history

Among the 150 children in the short-stature group, 35 (23.3%) had been diagnosed with GH deficiency. It was undergoing growth hormone therapy, while the remaining 115 children (76.7%) had no history of growth hormone treatment. A comparison between children receiving growth hormone therapy and those who were not revealed no significant difference in IL-6 or IL-8 levels with p-value =0.35 and p-value =0.42, respectively, indicating that elevated levels of these inflammatory cytokines may occur regardless of growth hormone treatment. However, the mean IGF-1 level in the treatment group was significantly higher than in the untreated group (p<0.001), underscoring the positive effect of growth hormone therapy on IGF-1 production.

### Statistical analysis of data

The inflammatory cytokine measurements, hormonal assays, and genetic analyses were evaluated using appropriate statistical methods. Significant differences were observed between the short-stature and control groups for all major variables, including GH, IGF-1, IL-6, IL-8, CRP and serum albumin levels, with all p-values being less than 0.05. Additionally, Pearson's correlation analysis confirmed a negative correlation between IL-6 and IGF-1 levels (r =-0.45, p<0.001). Genetic analysis of the GHR gene revealed a significant difference in mutation frequencies between the short stature and control groups (p<0.001), and children with GHR mutations had notably lower IGF-1 levels than those without mutations (p<0.01).

## Discussion

This study explored the underlying factors contributing to short stature in children, focusing on disturbances in the GH and IGF-1 axis, inflammation, and genetic mutations in the GHR gene. The findings revealed significantly reduced GH and IGF-1 levels in children with short stature compared to healthy controls, indicating dysfunction within this growth-regulating pathway. Additionally, inflammatory cytokines, particularly IL-6 and IL-8, were significantly elevated, suggesting that systemic inflammation may impair growth. Genetic testing showed a higher prevalence of GHR mutations among children with short stature, especially in exons 6 and 7, which are essential for receptor function. Children with these mutations also had lower IGF-1 levels, consistent with growth hormone insensitivity. The observed moderate inverse correlation between IL-6 and IGF-1 (r=-0.45) further supports a potential link between inflammatory pathways and GH/IGF-1 axis disruption.

These findings align with existing studies on growth disorders. Savage et al. [20] demonstrated that GH and IGF-1 deficiencies are primary contributors to impaired linear growth, consistent with our findings. Similarly, Chen et al. [21] found elevated IL-6 in children with delayed growth, supporting the role of chronic inflammation in growth failure. Cirillo et al. [22] further confirmed that IL-6 can interfere with GH signalling and IGF-1 synthesis, providing a mechanistic basis for our results. In our study, the elevated cytokines and decreased IGF-1 levels in children with short stature suggest that inflammation may either suppress IGF-1 directly or induce GH resistance through pathways such as SOCS3 activation, which inhibits GH receptor signalling.

Importantly, we observed that 23.3% of the children with short stature were receiving GH therapy. Subgroup analysis showed that while GH-treated children had significantly higher IGF-1 levels than untreated children (p<0.001), IL-6 and IL-8 levels did not differ between the two groups (p=0.35 and p = 0.42, respectively). This suggests that persistent inflammation may not be mitigated by GH therapy alone and could contribute to partial treatment resistance. These findings are consistent with previous literature indicating that inflammation may impair GH responsiveness at the receptor or post-receptor level [10] [22] [23] [24].

The moderate strength of the IL-6/IGF-1 correlation also suggests that additional factors may influence IGF-1 levels. Nutritional status, for instance, is a well-known modulator of growth, and serum albumin, while included in our analysis, can reflect both inflammatory status and malnutrition. Although we used albumin to assess systemic inflammation, we acknowledge its limitations in distinguishing these two conditions. Future studies should include more specific nutritional biomarkers such as prealbumin or transferrin to isolate the effects of malnutrition from inflammation better.

In terms of genetic analysis, the 14.7% prevalence of GHR mutations in our short-stature group is notably higher than typical population estimates. These mutations may reflect both known pathogenic variants and potentially novel ones, although functional validation was beyond the scope of this study. Compared to rare disorders like Laron syndrome, where GHR mutations are well-characterised and often homozygous, our findings suggest that heterozygous or milder variants may also impair growth through partial GH resistance [25]. Future studies should include functional assays and whole-gene sequencing to characterise the nature and impact of these variants.

Recent studies have further expanded our understanding of the inflammatory modulation of the GH/IGF-1 axis. A previous study demonstrated that children with systemic juvenile idiopathic arthritis treated with tocilizumab, an IL-6 receptor antagonist, showed improvement in growth velocity, even in the absence of GH therapy [26]. This supports the idea that IL-6 blockade may directly enhance growth by alleviating inflammatory suppression of GH signalling. Similarly, another article studied growth-impaired children with chronic inflammatory bowel disease and found that anti-TNF-therapy led to partial normalisation of IGF-1 levels and catch-up growth [27], suggesting that systemic cytokine burden directly contributes to growth hormone resistance regardless of underlying disease aetiology.

In the field of genetics, recent sequencing-based studies have uncovered a broader spectrum of GHR variants than previously recognised. A study in 2022 reported that among children with idiopathic short stature, some carried functionally relevant variants in GH signalling genes [28]. Another study emphasised that mild or partial GH insensitivity may account for a significant subset of growth disorders previously labelled as idiopathic [29]. These findings support our observation that a substantial proportion of children with short stature - 14.7% in our cohort - harbour GHR mutations, which may act independently or synergistically with inflammatory mechanisms to impair growth outcomes.

While this research provides meaningful views, it is not without limitations. Being a cross-sectional study, causality cannot be firmly established between inflammation, hormonal disruption, and short stature. Although our sample size was adequate, it may not represent the full spectrum of growth failure etiologies in children. Moreover, our focus on GHR mutations excluded other potentially relevant genetic pathways. We also recognise that the inflammatory marker panel was limited and that albumin alone may not capture nutritional contributions accurately.

To build upon these findings, future studies should implement longitudinal designs and include broader genetic and biochemical profiling. In addition to expanding mutation screening to other components of the GH/IGF-1 axis, evaluating newer markers of nutritional and inflammatory status may enhance understanding of these interactions. Clinical trials investigating the potential benefits of anti-inflammatory therapies, such as IL-6 blockade, alone or in combination with GH therapy, could reveal novel therapeutic strategies for children who show inadequate growth response despite hormone supplementation. Finally, incorporating IL-6 and IL-8 testing into the diagnostic workup of idiopathic short stature may help identify children with inflammation-driven growth suppression who could benefit from more targeted management approaches.

This study deepens our understanding of the hormonal, inflammatory, and genetic interplay in pediatric short stature and contributes valuable evidence toward more personalised and effective treatment paradigms.

## Conclusion

This research highlights that pediatric short stature results from a multifactorial interplay involving GH/IGF-1 axis dysfunction, elevated inflammatory cytokines (such as IL-6 and IL-8), and genetic mutations in the GHR gene. The findings suggest that both hormonal imbalances and persistent inflammation contribute to impaired growth and that inflammation may persist despite GH therapy, potentially leading to treatment resistance. These insights emphasise the need for a more comprehensive diagnostic approach that considers endocrine, inflammatory, and genetic factors. Specifically, we recommend incorporating IL-6 and IL-8 testing into the clinical evaluation of children with idiopathic short stature to identify inflammation-driven cases better. Further longitudinal and interventional studies are warranted to elucidate these molecular pathways and develop targeted therapies that can improve growth outcomes in affected children.

## Dodatak

### Conflict of interest statement

All the authors declare that they have no conflict of interest in this work.
